# Isolation syndrome in children: Clinical and psychological aspects in the context of COVID-19

**DOI:** 10.1192/j.eurpsy.2021.1772

**Published:** 2021-08-13

**Authors:** M. Ivanov, V. Kotlyarov, G. Kozlovskaya, L. Kremneva, M. Kalinina, T. Krylatova

**Affiliations:** Department Of Child Psychiatry, Federal State Budgetary Scientific Institution “Mental Health Research Center”, Moscow, Russian Federation

**Keywords:** COVID-19, pandemic, Deprivation, hildren

## Abstract

**Introduction:**

In connection with the COVID-19 pandemic, in order to prevent the spread of coronavirus infection, a lockdown was introduced everywhere in the Russian Federation, the main psychological feature of which is social deprivation - the deprivation or restriction of social habitual real interactions.

**Objectives:**

Describe clinical and psychological manifestations in children in the context of COVID-19 pandemic lockdown.

**Methods:**

During the period from June to September 2020, parents of 108 children of early, preschool and school age applied for advice. Children are divided into two groups: I -children with previously established mental disorders; II - children who have not previously been observed for developmental disorder by a psychiatrist.

**Results:**

Parents of children applied for counselling with complaints of psychological, neurotic and psychosomatic symptoms that were present in children during the period of lockdown. It was found that the active use of electronic gadgets, an abundance of alarming information from the media, can cause disturbances in the family system and become an additional risk factor in the developmental disorders and an increase in the existing psychopathological symptoms in children. However, in young children with normative development and in some children with autism spectrum disorder, the phenomenon of social isolation did not reveal any pronounced changes in the mental state towards deterioration; on the contrary, in a number of cases there is a weakening of previously manifested deviations, apparently associated with increased communication with the loved ones and increased parental attention.

**Conclusions:**

Clinical and psychological services should offer preventive support to the entire family.

**Disclosure:**

No significant relationships.

